# Supplementary material for: WordCommentsAnalyzer: A windows software tool for qualitative research
Source: F1000Res. 2018 Sep 4;7:536. Originally published 2018 May 3. [Version 2] doi: 10.12688/f1000research.14819.2 (PMC6137411; doi:10.12688/f1000research.14819.2)
Supplement: Supplementary file 3 [file f1000research-7-17614-s0002.tgz › b2a79496-4ada-47c4-9682-a99ce867836d.zip!/Eight Iranian Health Journals/Journal of Knowledge and Health_6.docx]

© Journal of Knowledge and Health. This work is licensed under CC BY 4.0 (https://creativecommons.org/licenses/by/4.0/).

Volume & Issue: دوره 11، شماره 4: 1395

Title: ارزیابی دو روش کونژوگاسیون کپسول پلی‌ساکاریدی هموفیلوس آنفلوانزا تیپ b با وزیکول غشاء خارجی نیسریا مننژیتیدیس سر و گروه B

http://knh.shmu.ac.ir/index.php/site/article/view/1434

مقدمه: هموفیلوس آنفلوانزا تیپ b (Hib) یکی از علل اصلی مننژیت در نوزادان و کودکان در کشورهای در حال توسعه است و هر ساله حداقل 3 میلیون مورد بیماری ناشی از Hib در سراسر جهان ایجاد می‌شود. کپسول Hib، عامل اصلی بیماری‌زایی می‌باشد. کپسول پلی‌ساکارید کونژوگه شده به پروتئین حامل در پیشگیری از عفونت‌ها مؤثر است. واکسیناسیون علیه Hib تاکنون در برنامه واکسیناسیون معمول کشور قرار نگرفته است. هدف از این مطالعه، دستیابی به یک روش کارآمد جهت تولید واکسن کونژوگه علیه مننژیت ناشی از هموفیلوس آنفلوانزا است. مواد و روش‌ها: در این تحقیق، مشتق پلی‌ساکارید Hib (PRP) در حضور 1-اتیل-3-(3-دی متیل آمینو پروپیل) کربودی‌ایمید (EDAC) با وزیکول غشاء خارجی (OMV) نیسریا مننژیتیدیس گروه B کونژوگه شد. دو روش با واسطه آدیپیک اسید دی‌هیدرازید (ADH) به‌عنوان فاصله‌گذار به‌کار برده شد. پلی‌ساکارید به‌صورت جداگانه با سیانوژن بروماید (CNBr) و  EDAC فعال شد. در روش اول، ADH به PRP فعال شده با CNBr متصل و مشتق PRPCNBr AH تشکیل شد. در روش دوم، ADH به PRP فعال شده توسط EDAC متصل و PRPEDAC AH تشکیل شد. دو مشتق ایجاد شده توسطEDAC  به OMV کونژوگه شدند و PRPCNBr AH-OMV،  PRPEDAC AH-OMV تشکیل شدند. نتایج: این تحقیق نشان داد که بازده کونژوگه PRPCNBr AH-OMV بر حسب پروتئین متصل شده به پلی‌ساکارید 3/63% و برای PRPEDAC AH- 47% OMV. نتیجه‌گیری: متوسط بازده کونژوگاسیون توسط سیانوژن بروماید فعال‌کننده بیشتر از فعال‌کننده دیگری است. با این حال به مطالعه بیشتر در مورد روش‌های دیگر و مقایسه آنها با یکدیگر برای دستیابی به یک واکسن کونژوگه مؤثر، نیاز است.

Title: تعیین شاخص‌های عملکرد ریوی کارگران یک صنعت نساجی در استان قم در سال 1392

http://knh.shmu.ac.ir/index.php/site/article/view/1487

مقدمه: کار در برخي شرایط می‌تواند بر سلامتی شاغلین اثر سوء داشته باشد. نزدیک به نیمی از عوامل خطر مؤثر در بروز بیماری‌های مزمن انسدادی ریوی دارای منشأ شغلی می‌باشند و این درحالی است که همه این موارد قابل پیشگیری هستند. در این مطالعه به تعيين اثرات ریوی ناشی از مواجهه با گرد و غبار الیاف اکریلیک در یکی از صنایع نساجی استان قم در سال 1392 پرداخته شد. مواد و روش‌ها: اين مطالعه، از نوع مطالعات توصيفي ‌تحليلي است كه به مدت 4 ماه بر روي كارگران كارخانه نساجي لاله مهرگان انجام شده است. به‌منظور جمع‌آوری اطلاعات فردی و شغلی از پرسشنامه استفاده شد و براي تعیین ظرفیت‌های ریوی کارگران، آزمون عملکرد ریوی با همکاری یک پزشک متخصص طب کار انجام شد. شاخص‌های عملکرد ریوی مورد بررسی شامل FEV1/FVC، FEV1، FVC و FEF 25- 75% بود؛ جهت توصیف و تجزیه و تحلیل داده‌ها از آزمون‌هاي آماری t تست، ضریب همبستگی پیرسون و تجزيه و تحليل واریانس یک طرفه در محیط SPSS استفاده گردید. نتايج: نتايج نشان داد كه ظرفیت‌های عملکرد ریوی در سالن‌هاي مختلف اختلاف آماری معناداری با یکدیگر نداشتند. در بین شاخص‌های عملکرد ریوی، تنها اختلاف میانگین FEF 25- 75% در بین گروه شاهد و گروه‌های شغلی ریسندگی و بافندگی از نظر آماری معنی‌دار بود. براساس آزمون ضریب همبستگی پیرسون بین عملکرد ریوی و سابقه‌ی شغلی، سن، شاخص توده‌ی بدنی و استفاده از تجهیزات حفاظت تنفسی ارتباط معناداری یافت نشد (05/0<P). نتیجه‌گیری: براساس نتايج مطالعه، احتمال شروع مراحل اولیه اختلالات مربوط‌به انسداد راه‌های هوایی به‌ویژه راه‌های هوایی کوچک در کارگران سالن‌های بافندگی و ریسندگی بالا است لذا بررسی و اصلاح سیستم تهویه در سالن‌های تولید ضروری است.

Title: طراحی و به‌کارگیری روش LAMP در جهت تشخیص مولکولی استرپتوکوک پیوژنز

http://knh.shmu.ac.ir/index.php/site/article/view/1512

مقدمه: استرپتوکوکوس پیوژنز کلونیزه‌کننده پوست و گلو در بسیاری از انسان‌ها است. این باکتری علاوه بر عفونت‌های چرکی حاد، مسئول عوارض و بیماری‌های خودایمن بعد از عفونت شامل آرتریت واکنشی، گلومرولونفریت و تب روماتیسمی می‌باشد. روش استاندارد طلایی برای شناسایی استرپتوکوکوس پیوژنز، کشت است. اما به‌دليل زمان‌بر بودن روش مذکور و اهمیت تشخیص سریع آن، استفاده از روش‌های مولکولی مفید است. تکنیک LAMP روش نسبتاً جدیدی است که حساسیت و اختصاصیت بالایی در تشخیص عفونت‌های باکتریایی دارد. هدف از این مطالعه، استفاده از تکنیک LAMP براساس ژن ستوکروم اکسیداز (oxaA) برای شناسایی استرپتوکوکوس پیوژنز است. مواد و روش‌ها: روش LAMP با 6 پرایمر برای توالی خاص oxaA در استرپتوکوکوس پیوژنز طراحی شد. غلظت‌های مختلفی از سولفات منیزیم، دزوکسی نوکلئوتید تری‌فسفات و پرایمرها برای بهینه‌سازی روش استفاده گردید. نتایج: واکنش LAMP با غلظت‌های 10 uM از هر کدام از پرایمرهای LB، LF،F3 و B3، 40 uM از هر کدام از پرایمرهای FIP و BIP، 0.4mM dNTPs و 8mM MgSo4 طی 1 ساعت و در دمای 63 درجه سانتیگراد راه‌اندازی گردید. نتیجه‌گیری: روش LAMP در مقایسه با PCR روش سریعی برای شناسایی استرپتوکوکوس پیوژنز می‌باشد.

Title: بررسی تأثير تمرین بازتوانی قلبی بر بیان ژن ABCA 1 در سلول‌های PBMN مبتلایان به ایسکمی قلبی

http://knh.shmu.ac.ir/index.php/site/article/view/1523

مقدمه: آترواسکلروز مهمترین بیماری قلب و عروق و عامل اصلی مرگ و میر در دنیا است. ABCA1 مسئول اصلی تشکیل ذراتHDL بوده و به همین دلیل احتمالاً در پیشگیری و جلوگیری از گسترش آترواسکلروزیس نقش دارد. هدف از مطالعه حاضر بررسی تأثير یک دوره تمرین ورزشی بازتوانی قلبی بر بیان ژن ABCA1 سلول‌های PBMN می‌باشد. مواد و روش‌ها: 23 نفر از بیماران مرد مبتلا به گرفتگی عروق قلبی انتخاب و به‌صورت تصادفی ساده به دو گروه کنترل و تجربی تقسیم شدند. گروه تجربی به مدت 8 هفته و هر هفته سه جلسه به مدت یک ساعت تمرینات مقاومتی سبک با شدت 40 تا 60 درصد یک تکرار بیشینه انجام دادند. قبل از شروع و بعد از پایان تمرینات میزان m-RNA ABCA1 به روش RT-PCR اندازه‌گیری و اطلاعات به‌وسیله تحلیل کوواریانس در سطح 05/0<P تجزیه و تحلیل شد. نتایج: نتایج نشان داد که تمرینات ورزشی بازتوانی موجب افزایش معنی‌دار بیان ژن ABCA1 و عدم تغییر HDL، LDL در بیماران گرفتگی عروق قلب شده است (05/0≤P).نتیجه‌گیری: بر اساس یافته‌های تحقیق می‌توان نتیجه‌گیری نمود که تمرینات ورزشی مقاومتی سبک می‌تواند در بیان ژن ABCA1 سلول‌های تک هسته‌ای خون بیماران MI مؤثر بوده باعث بهبود عملکرد انتقال معکوس کلسترول شده و احتمالاً در پیشگیری از وقوع ایسکمی‌های مکرر مؤثر باشد.

Title: تأثير ترکیبی تمرین استقامتی و آتورواستاتین بر سطوح سرمی اینترلوکین - 10 پس از انفارکتوس تجربی میوکارد در رت‌ها

http://knh.shmu.ac.ir/index.php/site/article/view/1532

مقدمه: آتورواستاتین و تمرینات استقامتی ممکن است اثرات مثبت مضاعفی بر فرآیند التهاب پس از انفارکتوس میوکارد (MI) داشته باشند. بنابراین، پژوهش حاضر با هدف بررسی تأثير ترکیبی تمرین استقامتی و سه دوز داروی آتورواستاتین بر سطوح سرمی اینترلوکین - 10 (IL-10) پس از انفارکتوس تجربی میوکارد در رت‌ها انجام شد.  مواد و روش‌ها: 75 سر رت نر (با سن 10-8 هفته و با میانگین وزن 10 ± 230 گرم) به‌طور تصادفی به 9 گروه کنترل کاذب (شم)، کنترل، تمرین استقامتی، آتورواستاتین (5، 10 و 15 میلی‌گرم/کیلوگرم) و تمرین + آتورواستاتین (5، 10 و 15 میلی‌گرم/کیلوگرم) تقسیم شدند. MI به وسیله‌ی تزریق زیرجلدی ایزوپرنالین (150 میلی‌گرم/کیلوگرم) در دو روز متوالی القاء شد. مداخله دارویی و تمرین استقامتی، 2 روز پس از MI شروع و به مدت 4 هفته تداوم یافت. داده‌ها با استفاده از آزمون تحلیل واریانس یک طرفه در سطح 05/0>P تحلیل شدند. نتایج: تمرین استقامتی همراه با مصرف 10 میلی‌گرم/کیلوگرم آتورواستاتین به‌طور معني‌داری سطوح سرمی IL-10 را افزایش داد (001/0=P)، اما تأثير تمرین استقامتی به تنهایی، دوزهای مختلف داروی آتورواستاتین به تنهایی و ترکیب تمرین استقامتی با دوزهای 5 و 15 میلی‌گرم/کیلوگرم آتورواستاتین تأثير معني‌داری بر سطوح سرمی IL-10 نداشت. نتیجه‌گیری: به‌نظر مي‌رسد که تمرین استقامتی همراه با مصرف 10 میلی‌گرم/کیلوگرم آتورواستاتین بهترین ترکیبی است که مي‌تواند وضعیت التهابی پس از MI را بهبود بخشد.

Title: بررسی اثر انکپسوله‌سازی سلول‌های بنیادی اسپرماتوگونی در هیدروژل آلژینات در طی انجماد-ذوب

http://knh.shmu.ac.ir/index.php/site/article/view/1550

مقدمه: اسپرماتوژنز پستانداران از سلول‌های بنیادی اسپرماتوگونی (SSCs) منشاء می‌گیرد. درمان‌های ضد سرطان، اعم از پرتودرمانی يا شیمی‌درمانی، با هدف قرار دادن این سلول‌های پرتكثير می‌توانند باعث ناباروری شوند. در مردان بالغ مبتلا به سرطان، انجماد اسپرماتوزوئیدها قبل از شروع درمان راه حفظ باروری در طی روند درمان است؛ اما در پسران نابالغ به دلیل نبود اسپرماتوزوئید، انجام این کار مقدور نیست. هدف این مطالعه بررسی اثر آلژینات بر کاهش اثرات سوء انجماد-ذوب بر پتانسیل SSCs و حفظ این سلول‌ها در بچه‌های سرطانی است که تحت شیمی‌درمانی یا رادیوتراپی قرار می‌گیرند. مواد و روش‌ها: SSCs از بیضه موش‌های 6 روزه نابالغ Balb/C جداسازی شدند. خالص‌سازی به‌وسیله آنتی‌بادی‌های Thy-1 و c-kit به روش MACS انجام گرفت. این سلول‌ها در هیدروژل آلژینات انکپسوله و منجمد شدند. بعد از ذوب سلول‌ها تعیین درصد سلول‌های زنده انجام گرفت. پس از استخراج RNA و ساخت cDNA بررسی بیان ژن‌های Oct4، Sall4، Plzf، Dazl، Etv5، Bcl6b، Lin28 و Nanog به روش Real time-PCR RT صورت گرفت. برای آنالیز نتایج از نرم‌افزار آماری SPSS و برای تجزيه و تحليل بیان ژن از نرم‌افزار Rest استفاده گردید. نتایج: درصد سلول‌های زنده پس از انجماد در گروه سلول‌های انکپسوله در آلژینات نسبت‌به گروه‌ کنترل کاهش آماری معنی‌داری را نشان نمی‌دهد. در گروه انجماد با انکپسوله‌سازی در آلژینات بیان ژن‌های Lin28 و Sall4 افزایش‌یافته (0001/0P<، 0001/0P<) و بیان ژن Dazl کاهش‌یافته است (0001/0P<). نتیجه‌گیری: این مطالعه نشان می‌دهد که استفاده از آلژینات به‌عنوان یک داربست در حفاظت سرمایی SSCs می‌تواند برای حفظ پتانسیل بنیادی این سلول‌ها مؤثر باشد.

Title: مقایسه ارزیابی موفقیت پیوند سلولی در پارکینسون تجربی با سنجش هموسیستئین با علایم رفتاری

http://knh.shmu.ac.ir/index.php/site/article/view/1535

مقدمه: پارکینسون از بیماری‌های تخریبی مغز به‌شمار می‌رود. شواهد نشان می‌دهد که میزان هموسیستئین سرم در بیماری پارکینسون افزایش می‌یابد. در این مطالعه ارزیابی موفقیت پیوند سلولی با مقایسه سطح سرمی هموسیستئین و علایم رفتاری در پارکینسون تجربی القا شده با 6-هیدروکسی دپامین مورد بررسی قرار گرفت. مواد و روش‌ها: اين مطالعه تجربی در سال 1393 بر روی موش‌های صحرایی نر ویستار انجام شد. موش‌ها ابتدا تحت جراحي استرئوتاکسيک با تزریق 6-هیدروکسی دپامین به ناحیه دسته مغز جلویی میانی نیمکره راست پارکینسونی شده که با آزمون چرخش القاء شده با آپومورفین تأیید گردید. پس از آن موش‌ها تحت درمان با پیوند سلول‌های بنیادی قرار گرفته و اثر درمان بر این چرخش‌ها مجدداً بررسی شد. سطح کلی هموسیستئین قبل و شش هفته پس از تزریق 6 -هیدروکسی دپامین و ده هفته پس از پیوند سلولی اندازه‌گیری گردید. داده‌ها با آزمون‌های آماري تی زوجی و واریانس یک طرفه و توکی تحلیل شدند. نتايج: سطح کلی هموسیستئین در موش‌های پارکینسونی با شمار چرخش بالا، تغییر معنی‌داری نداشت. ولی ده هفته بعد از پیوند سلولی، سطح کلی هموسیستئین به میزان قابل ملاحظه‌ای کاهش یافت. نتیجه‌گیری: از آنجایی که ارتباط مستقیمی بین شدت چرخش‌های القاء شده با آپومرفین با درجه آسیب سلولی در هسته جسم سیاه وجود دارد، نتایج ما نشان مي‌دهند که مقادیر بالاتر هموسیستئین مي‌تواند نشان‌دهنده آسیب نورونی بیشتر در این هسته باشد.

Title: بررسی درصد لنفوسیت‌های Th1 در خون محیطی بیماران مبتلا‌به سندرم هایپر IgE اتوزومال مغلوب

http://knh.shmu.ac.ir/index.php/site/article/view/1577

مقدمه: سندرم هایپر IgE نقص ایمنی اولیه است که با افزایش استعداد ابتلا به طیف محدودی از عفونت‌های قارچی و باکتریایی به‌خصوص کاندیدا آلبیکنس و استافیلوکوک اورئوس همراه است. بررسی تغییرات زیر گروه‌های لنفوسیت‌ها در بیماران در یافتن پاتوژنز بیماری می‌تواند کمک‌کننده باشد. در این مطالعه درصد لنفوسیت‌های Th1 در بیماران مبتلا‌به سندرم هایپر IgE اتوزومال مغلوب مورد بررسی قرار گرفت. مواد و روش‌ها: در این مطالعه مورد-شاهدی شش بیمار مبتلا‌به سندرم هایپر IgE اتوزومال مغلوب و هفت فرد سالم به‌عنوان گروه کنترل که با گروه بیماران از لحاظ سن و جنس همسان‌سازی شده بودند، مورد مطالعه قرار گرفتند. پس از خونگیری وریدی، سلول‌های تک‌هسته‌ای خون محیطی جدا گردیدند و پس از تحریک سلولی و کشت 12 ساعت، درصد سلول‌های Th1 به روش فلوسایتومتری مورد سنجش قرار گرفتند. نتایج: نتایج حاصل از این مطالعه نشان داد که درصد سلول‌های Th1 در بیماران در مقایسه با گروه شاهد به‌طور معني‌داری کاهش یافته است (008/0P<). نتيجه‌گيري: با توجه به نتایج این مطالعه به‌نظر می‌رسد که تغییر درصد زیر گروه‌های لنفوسیت Th1 ممکن است نقش مهمی در پاتوژنز این بیماری داشته باشد و کاهش درصد این لنفوسیت‌ها مي‌تواند در افزایش استعداد ابتلا به عفونت‌های قارچی و باکتریایی سهیم باشد.

Title: ارتباط فرسودگی شغلی و حمایت اجتماعی در کارکنان شاغل در بخش فوریت‌های پزشکی شهرستان کاشان

http://knh.shmu.ac.ir/index.php/site/article/view/1527

مقدمه: فرسودگی شغلی سندرمی شایع در میان مشاغلی است که در آنها ارتباط انسانی مطرح است. در این میان کارکنان بخش فوریت‌های پزشکی به علت ماهیت شغل خود و مواجهه با تنش‌هاي گوناگون شغلی نسبت‌به ساير مشاغل آمادگي بيشتري براي درگيري با اين سندرم دارند. یکی از راه‌های کاهش فرسودگی شغلی، حمایت اجتماعی است. لذا مطالعه حاضر با هدف تعیین ارتباط فرسودگی شغلی و حمایت اجتماعی در کارکنان فوریت‌های پزشکی شهرستان کاشان انجام شد. مواد و روش‌ها: پژوهش حاضر یک مطالعه مقطعی از نوع همبستگی بود که در سال 1394 انجام شد. 92 نفر از پرسنل فوریت‌های پزشکی شهرستان کاشان که به‌روش سرشماری انتخاب شده بودند، در این پژوهش شرکت کردند. ابزارهای مورد استفاده شامل پرسشنامه اطلاعات دموگرافیک، پرسشنامه «فرسودگی شغلی» ماسلاچ و جکسون و پرسشنامه «مقیاس چندبعدی حمایت اجتماعی درک شده» زیمت و همکاران بود. پس از تکمیل پرسشنامه‌ها داده‌ها با استفاده از نرم‌افزار SPSS مورد تجزیه و تحلیل قرار گرفت. نتایج: بین 47 تا 63 درصد از واحدهای مورد پژوهش خستگی عاطفی، مسخ شخصیت و عدم موفقیت فردی را در سطح متوسط تجربه کرده بودند. بین بعد حمایت از طرف خانواده و حمایت از طرف دیگران (افراد مهم) با خستگی عاطفی و مسخ شخصیت ارتباط معناداری وجود داشت (05/0<P). بین نمره کل حمایت اجتماعی و نمره کل فرسودگی شغلی ارتباط معناداری مشاهده نشد (05/0>P). نتایج به‌دست آمده همچنین نشان داد که بین سن و وضعیت تأهل با فرسودگی شغلی ارتباط معنادار آماری وجود دارد (05/0<P). نتیجه‌گیری: باتوجه به میزان قابل توجه فرسودگی شغلی در بین کارکنان فوریت‌های پزشکی و تأثير مثبت حمایت اجتماعی به‌ویژه از طرف خانواده و افراد مهم در کاهش فرسودگی، افزایش میزان حمایت صورت گرفته از طرف این افراد می‌تواند در کاهش میزان فرسودگی نقش مهمی داشته باشد. همچنین تدوین راهکارهای حمایتی توسط مدیران می‌تواند محیط مناسبی را برای فعالیت حرفه‌ای با نشاط کارکنان بخش فوریت‌های پزشکی فراهم نماید.

Title: کشف الگوی وضعیت اهداکنندگان خون از طریق خوشه‌بندی: روشی برای افزایش کیفیت خدمات در مراکز اهدای خون

http://knh.shmu.ac.ir/index.php/site/article/view/1525

مقدمه: نياز فوري به خون و عدم جايگزين مناسب براي آن، ضرورت وجود الگویی برای کمک به پزشکان جهت ارايه خدمات درست به اهداکنندگان و مدیریت صحیح پایگاه خون را نشان مي‌دهد. پژوهش حاضر با هدف شناسایی الگویی برای تشخیص وضعیت اهداکنندگان خون صورت گرفته است. مواد و روش‌ها: پژوهش حاضر به روش پیمایشی- مقطعي و به‌صورت سرشماری صورت گرفته است. جامعه پژوهش متشکل از داده‌هاي سازمان انتقال خون بيرجند در ماه‌های خرداد تا شهریور 92 است که با مراجعه مستقيم پژوهش‌گر به سازمان و به‌صورت فایل اکسل تهیه گردید. جهت تحليل داده‌ها از نرم‌افزار Clementine12.0 استفاده شده است. در پژوهش حاضر ابتدا خوشه‌بندی Two-Step و سپس الگوريتم‌های C5.0،C&R Tree ، CHAID و QUEST اجرا گردیدند تا بهترین نسبت بین فیلدهای مختلف به‌دست آید. نتایج: مقدار صحت به‌دست آمده از اجرای الگوریتم‌های C5.0، C&R Tree، CHAID و QUEST به‌ترتيب 9998/0، 9960/0، 9930/0، 8913/0 مي‌باشد. مقادیر به‌دست آمده برای شاخص‌های حساسیت، شفافیت، صحت، دقت، شاخص F، میانگین هندسی، نرخ مثبت غلط، نرخ منفی غلط و نرخ خطا برای مدل C5.0 نشان‌دهنده عملکرد بهتر اين الگوريتم نسبت‌به سایرین مي‌باشد. تأثيرگذارترین شاخص‌ها در تولید مدل، دسته فشارخون، وضعیت اهدای خون و دمای بدن هستند. نتيجه‌گيري: مدل ارايه شده به پيش‌بيني سریع‌تر و دقیق‌تر وضعیت اهدای خون و نیز مدیریت صحیح پایگاه خون کمک مي‌نماید و مي‌تواند گامی مؤثر جهت استفاده کارآمد از خون اهدایی و کاهش هزینه‌های نگهداری خون محسوب گردد.
